# Association between water source and chronic gastrointestinal diseases in Chinese: A cross-sectional and longitudinal study

**DOI:** 10.3389/fpubh.2022.992462

**Published:** 2022-11-10

**Authors:** Hehua Zhang, Yang Xia, Qing Chang, Xiangsu Zhang, Yuhong Zhao

**Affiliations:** ^1^Clinical Research Center, Shengjing Hospital of China Medical University, Shenyang, China; ^2^Department of Clinical Epidemiology, Shengjing Hospital of China Medical University, Shenyang, China; ^3^International Education School, China Medical University, Shenyang, China

**Keywords:** gastrointestinal disease, chronic disease, water supply, tap water, cohort study

## Abstract

**Background:**

Gastrointestinal health is closely associated with the quality of the water supply. However, long-term associations between the water supply type and chronic gastrointestinal disease (CGD) are unclear.

**Method:**

The water supply was categorized as “tap-water” or “non-tap water” use. Changes in water source use were categorized into four types: “non-tap water both at baseline and in follow-ups,” “non-tap water at baseline and tap-water in follow-ups,” “tap-water at baseline and non-tap water in follow-ups,” or “tap-water at baseline and in follow-ups.” We explored the association between tap-water use (and changes therein) and the risk of CGD in a cross-sectional and longitudinal population study based on national cohort data from 2011 to 2018.

**Results:**

After the inclusion and exclusion process, 13,332 and 9,688 participants were included in the cross-sectional and longitudinal analyses, respectively. Tap-water use was associated with fewer CGD cases at baseline (OR = 0.98, 95% CI: 0.90, 1.07). Tap-water use at baseline was associated with significantly lower incidence of CGD in follow-ups (HR = 0.70, 95% CI: 0.70, 0.90). Compared with consistent non-tap water use in both baseline and follow-ups, switching from non-tap water to tap-water use in follow-ups was associated with a lower risk of CGD (HR = 0.79, 95% CI: 0.64, 0.97), tap water use at both baseline and in follow-ups was associated with a lower risk of CGD (HR = 0.72, 95% CI: 0.59, 0.88). The decreased risk of CGD followed a linear trend (*P*_fortrend_ < 0.01). Adjustment for indoor solid fuel use and outdoor air pollution exposure to PM_2.5_ did not change the association between tap water use and CGD.

**Conclusion:**

Tap water use was associated with a reduced risk of incident CGD. The results from this study should aid in effect assessment for water purification strategies and public decision support for gastrointestinal health management.

## Introduction

Chronic gastrointestinal diseases (CGD), represented by peptic ulcer disease, inflammatory bowel disease, and gastroesophageal reflux disease, are characterized by recurrent chest pain, dyspepsia, emesis, diarrhea, halitosis, heartburn, or acid regurgitation, which influence participation in daily activities, decrease overall life-quality, and are associated with severe comorbidities in later life (1, 2). The overall prevalence of CGD has been increasing over the past 10 years, according to the global disease burden in 2020, particularly in developing counties (3–5). The etiology of CGD is complex and involves interactions among the immune system, intestinal microbiome, genetic susceptibility, and various environmental factors (6–9). Previous studies have indicated that older age, family history, sex, consumption of certain food and drinks, Helicobacter pylori infection, antibiotic abuse, and air pollution are risk factors for CGD; involvement of other environmental factors may explain the increase in CGD prevalence (6, 10, 11).

Water supply is essential for life. Previous studies have found that low quality water sources in rural areas or developing countries may contain high levels of chemicals (such as nitrate and trihalomethanes) (12, 13), heavy metals (such as chromium) (14), and bacteria (such as Escherichia coli and Helicobacter pylori) (15) in reservoirs of organic microelements or microorganisms. Consequently, long term exposure to low quality water may result in digestive diseases, respiratory diseases, as well as bladder, kidney, colorectal, and hepatocellular cancers. The underlying mechanism might involve changes in the gut microbiota, inflammation, oxidative stress, and mutations (16). Recently, a quantitative microbial risk assessment in South China has indicated that the annual probability of infection and the corresponding disease burdens caused by Cryptosporidium and Giardia in directly consumed water exceeded the threshold set by the World Health Organization (17). Unchlorinated consumption of water is associated with outbreaks of gastrointestinal illness (18). Therefore, gastrointestinal health is closely associated with the quality of the water supply. Previous studies have concentrated on the short-term effects of water contamination on acute digestive diseases or tumor induction (8, 19). However, the association between long-term exposure to different water supply types and CGD is unclear. With the implementation of strategies for improving residential water quality in China over the past several decades, the ratio of use of tap-water (sterilized) to of non-processed ground water has increased. We hypothesized that tap-water use would be associated with a decreased risk of CGD. In this study, on the basis of national cohort data from 2011 to 2018, we explored the association between tap-water use (and changes therein) and the risk of CGD in a cross-sectional and longitudinal population study.

## Materials and methods

### Participants

**Figure 1:**
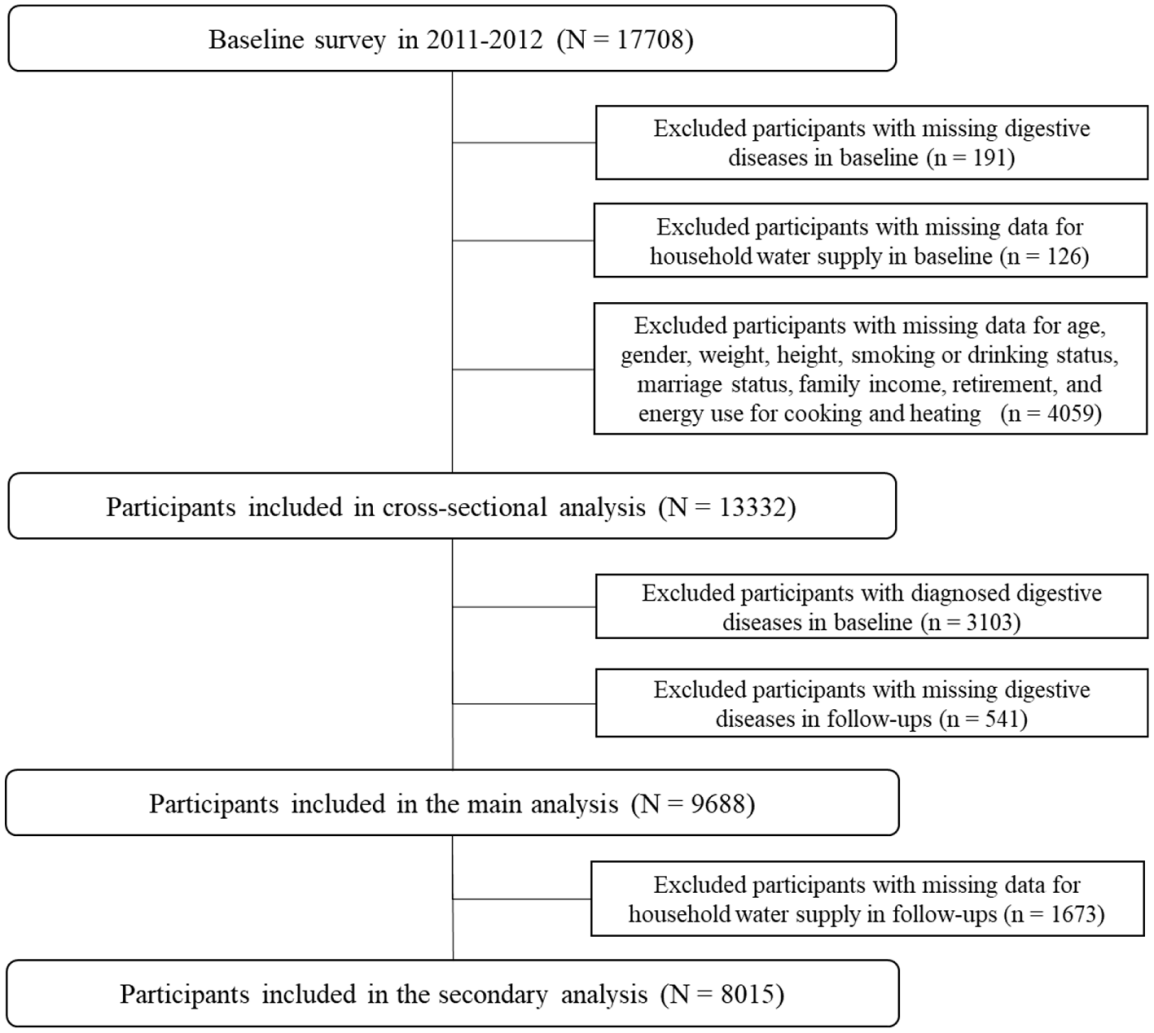
Selection process for included participants.

This study included participants from the China Health and Retirement Longitudinal Study (CHARLS), an ongoing national cohort with three waves of follow-ups in 2013, 2015, and 2018, covering 150 urban communities and 450 rural villages in 28 provinces. A total of 17,708 participants were included in the baseline survey, after exclusion of participants with missing CGD report data (*n* = 191); missing data on the household water supply (*n* = 126); and missing data on age, sex, weight, height, smoking or consumption status, marital status, family income, retirement, and energy source for cooking and heating (*n* = 4,059). A total of 13,332 participants were included in the cross-sectional analysis. After further exclusion of participants with diagnosed CGD at baseline (*n* = 3,103) and missing CGD in follow-ups (*n* = 541), 9,688 participants were included in the main analysis. Participants with missing data on water supply in follow-ups (*n* = 1,673) were excluded; consequently, 8,015 participants were included in the analysis of associations between switching of the water supply and the risk of CGD. The detailed selection process of participants is presented in Figure 1.

### Water source assessment

Water source data were acquired from a face-to-face questionnaire survey at baseline and in follow-ups in 2013, 2015, and 2018. Participants were asked, “Does your residence have processed tap-water?” Participants were categorized as having “tap-water” or “non-tap water” use at baseline and in follow-ups. Changes in water source use were categorized into four types: “non-tap water both at baseline and in follow-ups,” “non-tap water at baseline and tap-water in follow-ups,” “tap-water at baseline and non-tap water in follow-ups,” or “tap-water at baseline and in follow-ups.”

### CGD definition

Trained interviewers conducted face-to-face interviews with each participant or a household member. During the baseline survey, participants were asked whether they had been diagnosed with CGD (excluding hepatitis, liver cirrhosis, tumors, and cancer) by a physician. Meanwhile, during the follow-up surveys, participants without CGD at baseline were asked whether they had been diagnosed with GCD by a physician since the last interview 1 month, 1 year, or 2 years prior. The answer options were “yes,” “no,” and “do not know.” Participants who answered with “yes” were coded as having CGD or incident CGD during the baseline or follow-ups. Participants who answered with “do not know” were excluded.

### Assessment of other factors

#### Other environmental factors

Ambient air pollution exposure to particulate matter with a diameter < 2.5 μm (PM_2.5_) and indoor solid fuel use for cooking and heating were included as confounding factors in the assessment of associations with CGD in previous studies. The original PM_2.5_ data were obtained from the National Aeronautics and Space Administration. The average concentrations of PM_2.5_ at the city level from 1998 to 2010 were calculated in this study. Data on indoor fuel use for cooking and heating were collected in the baseline questionnaire survey. The use of energy sources such as “coal” and “crop residue/wood” was considered to indicate solid fuel use for heating or cooking, whereas the use of energy sources such as “solar,” “natural gas,” “marsh gas,” “liquefied petroleum gas,” or “electricity” was considered to indicate clean fuel use for heating or cooking.

#### Other confounding factors

Data on confounding variables, including age, sex, smoking and drinking behavior, education level, marital status, residential location, family income, chronic diseases, retirement status, weight, and height, were collected during the baseline face-to-face questionnaire survey. Marital status was categorized as married or single (widowed, never married, living alone, or divorced). BMI at baseline was defined as the participant's weight in kilograms (kg) divided by the square of the height in meters (m^2^). Residential locations were categorized as rural village or urban communities. Family income was categorized as higher income (≥4,113.93 yuan) or lower income (<4,113.93 yuan). Education level was categorized into three types: low (illiterate, did not finish primary school or finished elementary school), middle (did not finish or finished middle school, high school, or vocational school), and high (did not finish or finished college or above). Smoking and drinking behavior were separately divided into three types: never, ever, or current. Comorbidity numbers of other chronic diseases (including hypertension, dyslipidemia, diabetes or high blood glucose, heart attack, liver disease, kidney disease, emotional problems, arthritis, chronic lung diseases, asthma, or memory-associated diseases) were categorized into three types: zero, one, or two types of self-reported chronic diseases.

### Statistical analysis

Characteristics of the included participants were described according to their CGD status in follow-ups. Differences between groups were assessed with Chi-square tests (for categorized variables) or variance analysis (for numerical variables). Multiple logistic regression models and Cox proportional hazards regression models were used to explore the association between tap-water use and the risk of CGD in cross-sectional and longitudinal populations, respectively. The odds ratio (OR), hazard ratio (HR), and 95% confidence intervals (CIs) were calculated. Four models were used: the crude model, which did not included adjustment for any variables; model 1, which was adjusted for age, sex, and BMI; model 2, which was based on model 1 and was further adjusted for education level, smoking and alcohol consumption status, marital status, residential location, family income, social activity participation, number of comorbidities, and retirement status; and model 3, which was based on model 2 and further adjusted for indoor solid fuel use for cooking and heating, and outdoor air pollution exposure to PM_2.5_. We further explored the association between switching of tap-water use and the risk of CGD, and calculated the *P-*value for trend. Interaction and subgroup analyses according to age, sex, BMI, residential location, alcohol consumption status, smoking status, and marital status were conducted. Water and air pollution are both important environmental factors closely associated with health. For outdoor air pollution, we explored the association between long-term exposure to PM_2.5_ and incident CGD. For indoor air pollution exposure, we explored the association between indoor solid fuel use and incident CGD. All analyses were conducted in SAS (version 9.4; SAS Institute Inc., Cary, NC, USA). *P*-values were two-tailed, and associations were considered statistically significant when the *P*-value was lower than 0.05.

## Results

### Descriptive analysis

A total of 13,332 participants were included in the cross-sectional analysis. Participant characteristics are presented in Supplementary Table S1. Participants with diagnosed CGD tended to be younger (*P* = 0.01), be female (*P* < 0.0001), be married or living together (*P* < 0.01), be living in rural villages (*P* < 0.0001), have lower family income (*P* < 0.0001), have middle (*P* < 0.01) or high education (*P* < 0.01) level, and have higher rates of solid fuel use for heating (*P* < 0.0001) and cooking (*P* < 0.0001).

A total of 9,688 participants were included in the longitudinal analysis. Table 1 shows the characteristics of the participants included in longitudinal analysis according to GCD incidence at follow-ups. A total of 1,061 (31.82%) participants had incident CGD during the follow-ups (mean follow-up period of 6.28 years). The density of incident CGD in this cohort study was 17.43 per 1,000 person years. Participants with incident GCD tended to be younger (*P* = 0.01), be female (*P* < 0.0001), have lower BMI (*P* < 0.05), have non-tap water use (*P* < 0.0001), and have indoor solid fuel use for heating (*P* < 0.01).

**Table 1:** Characteristics of the included participants according to GCD incidence in follow-ups.

**Characteristics**	**GCD status**		***P-*value^a^**
	**Yes (*n =* 1,061)**	**No (*n =* 8,627)**	
Age, year	57.84 (57.26, 58.42)^b^	58.63 (58.43, 58.84)	0.01
Male	425 (40.06)^c^	4,199 (48.67)	<0.0001
BMI, kg/m^2^	23.44 (23.2, 23.68)	23.69 (23.61, 23.78)	<0.05
Married	929 (87.56)	7,537 (87.37)	0.86
Rural village	686 (64.66)	5,389 (62.47)	0.16
Higher income^d^	158 (14.89)	1,460 (16.92)	0.09
Social activity	486 (45.81)	4,131 (47.88)	0.20
Retired	261 (24.60)	2,258 (26.17)	0.27
**Education level** ^**e**^			
Low	202 (19.04)	1,841 (21.34)	0.08
Middle	384 (36.19)	3,038 (35.22)	0.53
High	475 (44.77)	3,748 (43.44)	0.41
**Smoking status**			
Never	670 (63.15)	5,201 (60.29)	0.07
Ever	89 (8.39)	733 (8.50)	0.91
Current	302 (28.46)	2,693 (31.22)	0.07
**Drinking status**			
≥1time per month	224 (21.11)	2,269 (26.30)	<0.001
<1time per moth	77 (7.26)	679 (7.87)	0.48
Never	760 (71.63)	5,679 (65.83)	<0.001
**Comorbidities**			
None	378 (35.63)	3,731 (43.25)	<0.0001
One	348 (32.80)	2,756 (31.95)	0.57
≥Two	335 (31.57)	2,140 (24.81)	<0.0001
Tap water use	583 (54.95)	5,316 (61.62)	<0.0001
**Solid fuel use**			
For cooking	622 (58.62)	4,796 (55.59)	0.06
For heating	814 (76.72)	6,297 (72.99)	<0.01
PM_2.5_	33.58 (32.72, 34.43)	34.34 (34.04, 34.64)	0.10

BMI, body mass index; CGD, chronic gastrointestinal disease.

a Analysis of variance or chi-squared test.

b Least square mean (95% confidence interval) (all such values).

c Counts (percentages) (all such values).

d A family income higher than the median (4,114 RMB) was classified as higher income.

e Low, illiterate, did not finish primary school or finished elementary school; Middle, did not finish or finished middle school, high school, or vocational school; High, did not finish or finished college or above.

### Association between tap-water use and CGD

Table 2 shows the associations between tap-water use and CGD in cross-sectional and longitudinal analyses. In the cross-sectional analysis, the association between tap-water use and CGD at baseline was statistically non-significant (OR = 0.98, 95% CI: 0.90, 1.07). In the longitudinal analysis, tap-water use at baseline was associated with significantly lower incidence of CGD in follow-ups (HR = 0.70, 95% CI: 0.70, 0.90).'

**Table 2:** Association between tap-water use and CGD.

**Regression models**	**ORs and** **95%CI^$^**	**HRs and** 95%CI^*^
Crude model	0.84 (0.78, 0.92)	0.78 (0.69, 0.88)
Model1^a^	0.86 (0.79, 0.93)	0.79 (0.70, 0.89)
Model2^b^	0.95 (0.87, 1.03)	0.79 (0.70, 0.90)
Model3^c^	0.98 (0.90, 1.07)	0.80 (0.71, 0.92)

$ Multiple logistic regression in the cross-sectional analysis.

* Cox proportional hazards regression in the longitudinal analysis.

a Adjusted for age, sex, and BMI.

b Further adjusted for education level, smoking and alcohol consumption status, marital status, residential location, family income, social activity participation, number of comorbidities, and retirement status; based on model 1.

c Further adjusted for indoor solid fuel use for cooking and heating, and outdoor air pollution exposure to PM_2.5_; based on model 2.

### Association between switching tap-water use and CGD

Table 3 shows the association between switching tap-water use and CGD. Compared with participants with consistent non-tap water use at both baseline and follow-ups, switching from non-tap water to tap-water use in follow-ups was associated with a lower risk of CGD (HR = 0.79, 95% CI: 0.64, 0.97), tap water use at baseline and in follow-ups was associated with lower risk of CGD (HR = 0.72, 95% CI: 0.59, 0.88). The decreased risk of CGD followed a linear trend (*P*_fortrend_ < 0.01).

**Table 3:** Association between switching tap-water use and CGD.

**Cox proportional hazards regression**	**Non-tap water in both baseline and follow-ups**	**Non-tap water at baseline and tap-water in follow-ups**	**Tap water at baseline and non-tap water in follow-ups**	**Tap water in both baseline and follow-ups**	*P* for trend^*^
No. of participants	1,141	2,012	436	4,426	
No. of incident cases	154	223	46	427	
Crude model	Ref	0.81 (0.66, 0.99)	0.77 (0.56, 1.08)	0.70 (0.58, 0.85)	<0.001
Model1^a^	Ref	0.80 (0.65, 0.99)	0.77 (0.55, 1.06)	0.71 (0.59, 0.85)	<0.001
Model2^b^	Ref	0.79 (0.64, 0.97)	0.77 (0.55, 1.07)	0.71 (0.58, 0.86)	<0.001
Model3^c^	Ref	0.79 (0.64, 0.97)	0.78 (0.56, 1.09)	0.72 (0.59, 0.88)	<0.01

* Tests for linear trend were performed by entering the value of each category of water use as a continuous variable in the models.

a Adjusted for age, sex, and BMI.

b Further adjusted for education level, smoking and alcohol consumption status, marital status, residential location, family income, social activity participation, number of comorbidities, and retirement status; based on model 1.

c Further adjusted for indoor solid fuel use for cooking and heating, and outdoor air pollution exposure to PM_2.5_; based on model 2.

### Subgroup analysis

**Figure 2:**
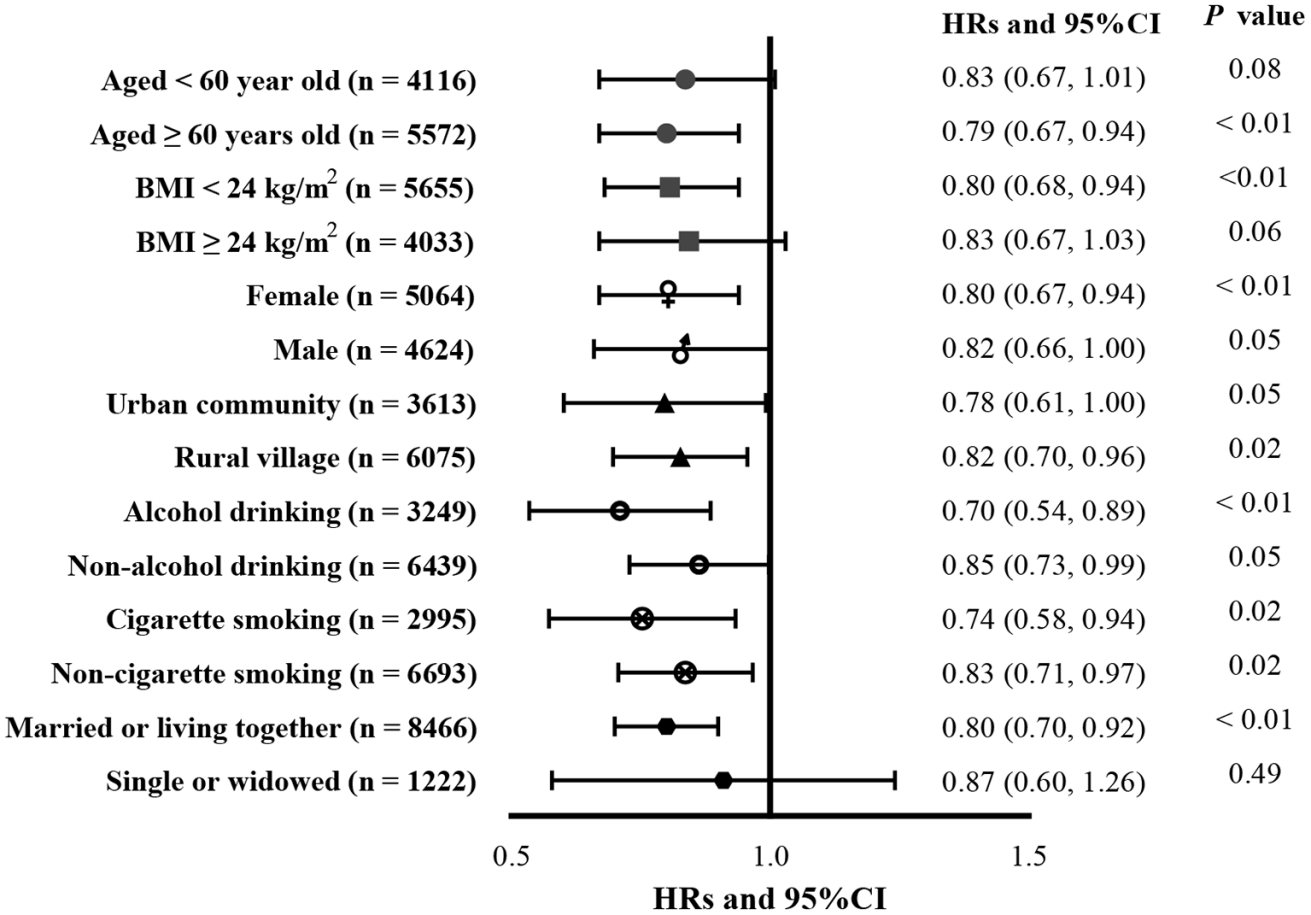
Subgroup analysis.

No interactions between tap-water use and other factors were found in this study (all *P*_forinteraction_ > 0.05). Associations between tap water use and incident CGD according to age, BMI, sex, living location, alcohol consumption, cigarette smoking, and marital status are presented in Figure 2. Tap water use was associated with a lower risk of incident CGD in participants over 60 years of age (HR = 0.79, 95% CI: 0.67, 0.94), participants with lower BMI (< 24; HR = 0.80, 95% CI: 0.68, 0.94), female participants (HR = 0.80, 95% CI: 0.67, 0.94), participants with alcohol consumption (HR = 0.70, 95% CI: 0.54, 0.89) and cigarette smoking (HR = 0.74, 95% CI: 0.58, 0.94), and participants with non-single marital status (HR = 0.80, 95% CI: 0.70, 0.92).

### Other environmental factors and CGD

Associations between air pollution from indoor solid fuel use and outdoor exposure to PM_2.5_ are shown in Supplementary Table S2. Indoor use of solid fuel for cooking (HR = 1.02, 95% CI: 0.88, 1.18) and heating (HR = 1.06, 95% CI: 0.90, 1.25) were both associated with elevated risk of incident CGD, but the association was not statistically significant. No association between outdoor air pollution exposure to PM_2.5_ and incident CGD was found.

## Discussion

In this cross-sectional longitudinal study, we found that sterilized tap water use was associated with a lower risk of CGD; the association was statistically significant only in the longitudinal analysis. Switching from non-tap water to tap water was also associated with a lower risk of CGD. Adjustment for indoor solid fuel use and outdoor air pollution exposure to PM_2.5_ did not change the association between tap water use and CGD.

Previous studies have reported the association between a contaminated water supply and the outcome of CGD. A case-control study in Italy has reported that contamination of municipal water by norovirus leads to the development of post-infectious irritable bowel syndrome and functional gastrointestinal disorders (20). A cohort study in Canada has reported similar results (21). Previous cohort or cross-sectional studies in Norway (22), Sweden (23), and America (24) have all reported that incidents (i.e., interruption, breaks, or maintenance work) in the water distribution systems were associated with increased risk of gastrointestinal disease among water recipients. One cross-sectional study in northern Canada has reported that an increase in bacteria in stored water for consumption was not a risk factor for acute gastrointestinal illness (25). The reason for this finding might have been that the effect of a contaminated water supply on CGD is time dependent. Only limited studies have reported the benefits of long-term quality improvement of the water supply on CGD. A cohort study in Sweden has reported that improved water treatment decreases the gastrointestinal disease burden in children 0–9 years old (26). An intervention study in Africa has reported that increasing the quality of the water supply markedly improved health outcomes of diarrhea incidence in rural areas (27). The longitudinal results in this study indicated that tap-water use or switching from non-tap water to tap water use was associated with a diminished risk of CGD, in accordance with previous longitudinal studies. However, the follow-up durations in previous studies were shorter than that in this study, thus potentially preventing collection of data pertaining to more chronic gastrointestinal diseases, such as peptic ulcers. Previous studies have reported that air pollution exposure was associated with an elevated risk of gastrointestinal cancers but not peptic ulcer disease (28, 29). We found that only air pollution of indoor solid fuel use was associated with an increased risk of CGD. The effects of PM_2.5_ exposure from outdoor air pollution on CGD should be explored in further research.

The mechanisms underlying the association between contaminated water and gastrointestinal disease may include: a. a potential association between the content and ratios of micro-elements in contaminated water for consumption (such as chlorides, nitrates, phosphates, chlorates, sulfates, free chlorine, sodium, magnesium, potassium) and the presence of Helicobacter pylori, thus resulting in peptic ulcer (30); b. more heavy metals in consumed water may increase inflammation or immune responses in gastrointestinal micro-environments (16, 31); c. drinking water quality is closed associated with the gut microbiota composition, thus affecting gastrointestinal health and function (32). The possible reason why tap water decreases the risk of CGD might be due to the lower content of ionic and organic microelements, heavy metals, bacteria, chemicals, and microorganisms, owing to the treatment processes of coagulation, precipitation, filtration, and disinfection (using perfluoroalkyl substances), according to the household consumption water standard in China (16, 33, 34) and the World Health Organization (35).

We found no interactions between tap-water use and the other factors of age, BMI, sex, residential location, alcohol consumption or cigarette smoking, or marital status. Subgroup analysis showed that the protective effect of tap-water use was present in participants who were above 60 years of age, had lower BMI, were either male or female, were living in urban communities and rural villages, or had behaviors of alcohol consumption and cigarette smoking, in accordance with previous studies indicating that fluoridated tap water provides more health benefits than other water supply types (36, 37). Although the rate of tap-water access is increasing in rural areas and covers all urban areas, most residents consumed more bottled than tap-water, although tap-water is a low-cost and ecologically friendly resource. Further recommendations of tap water use for consumption should be made.

This study is the first and largest cohort study reporting the long-term effect of tap-water use and switching tap water use on CGD in a Chinese population. Our results indicated that long-term tap water use, as well as switching from non-tap water use to tap water use, was beneficial for the prevention of CGD; this effect was independent of other environmental factors including age, BMI, sex, education level, smoking and alcohol consumption status, marital status, residential location, family income, social activity participation, number of comorbidities, retirement status, and air pollution. However, because this was an observational study, it had several limitations. First, we used self-reported data on CGD diagnosed by a physician as the study outcome. Although the follow-up study involved door-to-door interviews, and the positioning of participants' home addressees at baseline and the self-reported disease were reliable and had limited missing data, further studies should more precisely classify CGD. Second, we could not assess the changes in cofounding factors from baseline to follow-ups, as is inevitable in epidemiological studies. Third, beyond the available covariates in this study, we could not exclude the possibility that other potential factors (such as hot water usage or home water filter devices) might potentially have affected the association between tap water use and CGD. Forth, we could not exclude those participants with CGD incident may occur before the switch of tap water use for the reason that we did not record time information of tap water use or CGD incidence in follow-ups. Finally, because the CHARLS study mainly included adults who were middle age or older in China, the results of this study cannot be extended to all ages or populations in other counties.

## Conclusion

Tap water use was associated with a decreased risk of incident CGD. Switching from non-tap water to tap water use, compared with consistent non tap water use, was associated with a lower risk of incident CGD. The association between tap water use and CGD was independent of other environmental factors including air pollution. The results of this study should aid in effect assessment for water cleaning strategies and public decision support for gastrointestinal health management (38).

## Data availability statement

The datasets presented in this study can be found in online repositories. The names of the repository/repositories and accession number(s) can be found below: http://charls.pku.edu.cn/index.htm.

## Ethics statement

The studies involving human participants were reviewed and approved by Ethics Committee of Peking University. The patients/participants provided their written informed consent to participate in this study.

## Author contributions

HZ and YX conducted the analysis process and wrote the paper. QC and XZ reviewed and revised the paper. YZ designed and revised the study. All authors contributed to the article and approved the submitted version.

## Funding

This work was supported by the National Natural Science Foundation of China (No. 82103791), the Natural Science Foundation of Liaoning Province (No. 2020MS172), and the 345 Talent Project of Shengjing Hospital of China Medical University (No. M0294).

## Conflict of interest

The authors declare that the research was conducted in the absence of any commercial or financial relationships that could be construed as a potential conflict of interest.

## Publisher's note

All claims expressed in this article are solely those of the authors and do not necessarily represent those of their affiliated organizations, or those of the publisher, the editors and the reviewers. Any product that may be evaluated in this article, or claim that may be made by its manufacturer, is not guaranteed or endorsed by the publisher.

## Abbreviations

CGD: chronic gastrointestinal disease
OR: odds ratio
HR: hazard ratio
CI: confidence interval
PM_2.5_: particulate matter with a diameter <2.5 μm
BMI: body mass index.
