# Supplementary material for: Association between water source and chronic gastrointestinal diseases in Chinese: A cross-sectional and longitudinal study
Source: Front Public Health. 2022 Nov 10;10:992462. doi: 10.3389/fpubh.2022.992462 (PMC9685615; doi:10.3389/fpubh.2022.992462)
Supplement: Supplementary file 1 [file Table_1.DOCX]

**Table S1**. Characteristics of participants included in the cross-sectional analysis

| Characteristics | GCD status | | *P* value ^a^ |
| --- | --- | --- | --- |
|  | Yes (n = 3103) | No (n = 10229) |  |
| Age, year | 58.31 (57.96, 58.65) ^b^ | 58.82 (58.63, 59.01) | 0.01 |
| Male | 1323 (42.64) ^c^ | 4906 (47.96) | < 0.0001 |
| BMI | 23.01 (22.87, 23.15) | 23.63 (23.56, 23.71) | < 0.0001 |
| Married | 2758 (88.88) | 8877 (86.78) | < 0.01 |
| Rural village | 2104 (67.81) | 6304 (61.63) | < 0.0001 |
| Higher income ^d^ | 384 (12.38) | 1698 (16.60) | < 0.0001 |
| Social activity | 1428 (46.02) | 4864 (47.55) | 0.14 |
| Retired | 791 (25.49) | 2790 (27.28) | 0.05 |
| Education level ^e^ |  |  |  |
| Low | 665 (21.43) | 2153 (21.05) | 0.65 |
| Middle | 1167 (37.61) | 3577 (34.97) | < 0.01 |
| High | 1271 (40.96) | 4499 (43.98) | < 0.01 |
| Smoking status |  |  |  |
| Never | 1931(62.23) | 6156 (60.18) | 0.04 |
| Ever | 268 (8.64) | 905 (8.85) | 0.72 |
| Current | 904 (29.13) | 3168 (30.97) | 0.05 |
| Drinking status |  |  |  |
| ≥1time per month | 660 (21.27) | 2611 (25.53) | < 0.0001 |
| ＜1time per moth | 244 (7.86) | 809 (7.91) | 0.93 |
| Never | 2199 (70.87) | 6809 (66.57) | < 0.0001 |
| Comorbidities |  |  |  |
| None | 835 (26.91) | 4283 (41.87) | < 0.0001 |
| One | 1034 (33.32) | 3256 (31.83) | 0.12 |
| ≥ two | 1234 (39.77) | 2690 (26.30) | < 0.0001 |
| Tap water use | 1771 (57.07) | 6260 (61.20) | < 0.0001 |
| Solid fuel use |  |  |  |
| For cooking | 1948 (62.78) | 5669 (55.42) | < 0.0001 |
| For heating | 2416 (77.86) | 7452 (72.85) | < 0.0001 |
| PM_2.5_ | 33.83 (33.34, 34.33) | 34.18 (33.91, 34.46) | 0.23 |

Abbreviations: BMI, body mass index; CGD, chronic gastrointestinal disease.

^a^ Analysis of variance or chi-squared test.

^b^ Least square mean (95% confidence interval) (all such values)

^c^ Counts (Percentages) (all such values)

^d^ A family income higher than the median (4,114 RMB) was classified as higher income

^e^ Duration of education ≤ 6 years, > 6 years and ≤ 12 years, < 12 years were categorized as low, middle, or high education level.

**Table S2.** Association between air pollution of indoor solid fuel use and outdoor exposure of PM_2.5_

|  | solid fuel use for cooking | solid fuel use for heating | outdoor exposure of PM2.5 |
| --- | --- | --- | --- |
| Crude model | 1.11 (0.98, 1.25) ^d^ | 1.17 (1.01, 1.34) | 1.00 (0.99, 1.00) |
| Model1 ^a^ | 1.09 (0.96, 1.23) | 1.14 (0.99, 1.32) | 1.00 (0.99, 1.00) |
| Model2 ^b^ | 1.08 (0.95, 1.24) | 1.13 (0.97, 1.32) | 1.00 (0.99, 1.00) |
| Model3 ^c^ | 1.02 (0.88, 1.18) | 1.06 (0.90, 1.25) | 1.00 (0.99, 1.00) |

^a^ Adjusted for age, gender, and BMI.

^b^ Further adjusted for education level, smoking and alcohol drinking status, marriage status, residential location, family income, social activity participation, number of comorbidities, and retirement status based on model1.

^c^ Further adjusted for indoor solid fuel use for cooking and heating, and outdoor air pollution exposure of PM_2.5_ based on model 2.

^d^ Hazard ratio and 95% confidence interval calculated from Cox proportional hazards regression in the longitudinal analysis
